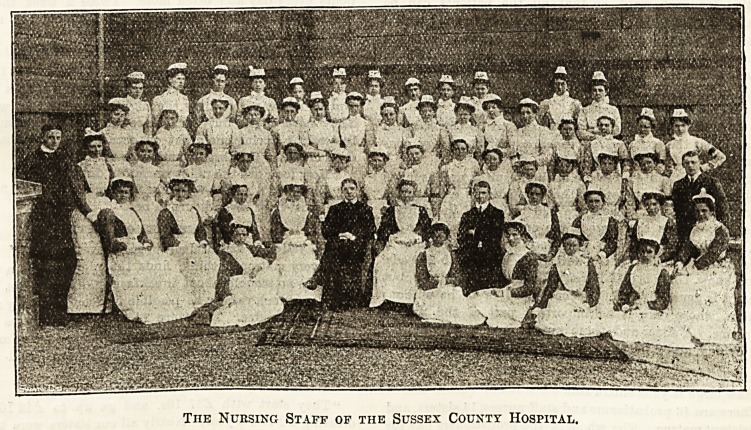# The Hospital. Nursing Section

**Published:** 1905-02-04

**Authors:** 


### The Hospital.

### Nursing Section.

Contributions for this Section of "THE HOSPITAL" should be addressed to the EDITOR, "THE HOSPITAL," NURSING SECTION, 28 & 29 Southampton Street, Strand, London, W.C.

No. 958.—VOL. XXXVII. *SATURDAY, FEBRUARY* 4, 1905.

### Notes on News from the Nursing World.

### INCREASING DEMANDS FOR QUEEN'S NURSES.

THE triennial meeting of the Queen's Commemoration Fund took place on Tuesday afternoon. Sir Dyce Duckworth, who presided, moved the adoption of the report and accounts for 1904. Having stated that during the year the sum of £2,250 had been handed by the committee to the Queen Victoria Jubilee Institute for Nurses, he said that an additional income of £2,000 per annum was needed by the council of the institute in order to maintain the present standard of efficiency and to meet the increasing demands for Queen's nurses throughout the country. The Hon. Sydney Holland, Mr. J. E. Greaves, Lord-Lieutenant for the County of Carnarvon, and Mr. Harold Boulton, M.V.O., were selected for recommendation to her Majesty for appointment as members of the Council of the Queen's Institute representing the subscribers.

### THE VALUE OF SEPARATE BEDROOMS.

IN an interview with our Commissioner, the matron of the Sussex County Hospital dwells on the value of separate bedrooms as against cubicles, which she says it is impossible to over-estimate. She finds that in addition to the measure of privacy which is secured to a nurse by the possession of a room of her own, the system conduces to the maintenance of discipline, and she thinks that it is almost incredible that anyone would propose to perpetuate cubicles in a new home. In all respects but one, the nurses' home at the Sussex County Hospital seems to be admirable. It is a pity that the whole of the nurses' bedrooms are not fitted with fireplaces. At any rate, we advise the projectors of new homes to provide them.

### TOO MANY NURSES IN THE TRANSVAAL.

ANOTHER warning note comes from South Africa, and this time from a different quarter. That the surplus of nurses in the Transvaal is not imaginary is evident from one fact in particular. In Johannesburg alone there are 800 nurses, and 200 are quite sufficient to supply the needs of the sick! Formerly Johannesburg was considered unhealthy, but now that the Town Council look after the sanitary arrangements and see that the streets are kept free from dust and dirt, pneumonia and enteric fever, which at one time were rife, are comparatively rare. Moreover, money is very scarce, so that maternity nurses get only fortnightly engagements, and mothers nurse their little ones themselves instead of employing a trained nurse, except in cases of great danger. Young nurses without capital especially should not venture to the large towns, where there are many temptations, for they cannot live on less than £10 a month, and during 1904 numbers of nurses have earned on an average not more than £5, whilst some have only obtained work for four days in as many months. Accordingly we repeat the advice to nurses which we have given before—not to emigrate to South Africa unless they have enough capital to live on for a year, or are going out to take up an assured position.

### FRICTION AT A DUBLIN WORKHOUSE HOSPITAL.

THERE was a long discussion last week at a meeting of the Guardians of the South Dublin Union respecting the report of the Visiting Committee of the Protestant Hospital. The gist of the report was that the committee, from evidence given at an inquiry touching the question of providing substitutes during the absence of nurses, came to the conclusion that the superintendent nurse was not capable of fulfilling her duties, and recommended that she should be superseded. This conclusion appears to have been due to allegations on the part of another nurse that meat was supplied to patients uncooked; that eggs were sold to patients; that the superintendent nurse refused to do her share of the extra work when one of the nurses was on leave; that she had a meeting one Monday evening at which there was singing and music, in consequence of which it was impossible to attend the patients in some of the wards; and that on the previous Saturday a member of the Salvation Army distributed tracts in one of the wards. It transpired, however, that the superintendent nurse was not allowed to be present at the inquiry when the most important evidence in support of these allegations was given; and the Guardians eventually unanimously decided to refer the report back to the committee requesting them to supply a copy to the superintendent nurse, ask for her observations on it, and then again inquire into the whole matter. We think that they have pursued a very proper course. That there is friction in the Protestant Hospital between the superintendent nurse and her subordinates is very clear, and we are afraid that it is due to petty causes, as well as to want of the kindly feeling which should always prevail amongst fellow workers. But the proposal to degrade the superintendent nurse, and to put in her place a junior and the chief witness against her, without affording her the fullest opportunity of stating her case, was manifestly unfair.

### COOKERY INSTRUCTION FOR NURSES.

AT the last meeting of the Bethnal Green Guardians a report was presented from the examiners of the London County Council, which stated that 16 of the nurses at the infirmary entered for the practical cookery examination, and that of these, 14 gained first-class, and two second-class marks. The examiner added that the nurses worked neatly and well, and appeared to be keenly interested in what they were doing. Several of the guardians expressed their satisfaction with the report, but Dr. Style protested against the lessons being given, and said that a nurse's duty was "to tend the sick and dying, and not to cook daintily." As in our opinion a portion of the duty of tending the sick is to see that the food prepared for them is of an apetitising and suitable kind, we think that cookery instruction should be given wherever it can be. In private nursing it is almost indispensable, and as so many probationers in hospitals and workhouse infirmaries contemplate taking up private nursing, the inclusion of cookery lessons in the curriculum is obviously an advantage likely to be appreciated.

### NIGHT DUTY AT FROME.

An inspector of the Local Government Board has pointed out to the Frome Guardians that the nursing system is defective, there being only two nurses to look after 40 patients, many of whom are bedridden. In his view it is not reasonable that the nurses should be expected to perform both day and night duty. The reply of the guardians is that the head nurse is supposed to be on duty by day and the assistant nurse by night, and that they are always ready to grant an extra nurse for night duty whenever the medical officer says that one is required. Whatever may be the opinion of the Frome Guardians, or of their medical officer, provision should be made for the regular attendance of a nurse during the night. It should not be a matter of supposition, but an indisputable fact.

### GUARDIANS AND CONTRIBUTIONS TO NURSING ASSOCIATIONS.

An interesting proposal is before the Chesterton Guardians, and will come up for further consideration at the next meeting. The Finance Committee recommended the Guardians to offer to contribute a sum equal to a halfpenny rate, which will produce about £46, for each medical officer's district, towards the support of a fully-qualified nurse in each such district, on condition that voluntary contributions be raised in each district to provide the balance required for the support of the nurse. As a temporary measure they advise that £25 per annum be paid to the Cambridge District Nursing Association towards the support of a nurse at Chesterton, with the stipulation that assistance be given to pauper patients. There are seven medical districts, and the committee think that instead of the Guardians contributing as they had been asked to any special association, they should take a wider view and set up a system applicable to the whole of the Union. The scheme appears to us to be one that would be a substantial help to the Nursing Associations, as it would mean the contribution of half the yearly salary of a trained nurse. It would also be for the benefit of the people just above the class who receive relief, as well as for outdoor patients, and it would be no appreciable burden to the ratepayers.

### AN OVER-WORKED NURSE AT EPSOM.

There was one point upon which all who attended the annual meeting of the Epsom District Nursing Association were agreed, namely, that a single nurse is not sufficient for the needs of 11,000 inhabitants. We endorse that conclusion, and hope that the sub-committee, which has been formed in order to decide upon a scheme for providing a second nurse will arrive at a satisfactory solution of the problem. Last year the single nurse paid 3,406 visits and attended 117 cases. Hitherto, a *locum tenens* has not been engaged during her badly-needed holiday. This is not, in any event, to occur again. But it should not be difficult to obtain in the large and prosperous town of Epsom sufficient money to pay the regular salaries of two nurses.

### THE PRIMROSE LEAGUE AND QUEEN'S NURSES.

Under the auspices of the Central Habitation of the Primrose League at Sheffield, a concert was held last week in aid of the local branch of the Queen's Jubilee Institute. This was the first of a series of entertainments organised by the League for the benefit of various charities, and we hope that the example thus set will be followed in other towns. Sir Howard Vincent, M.P., who was present on the occasion, expressed himself rejoiced to see many political opponents of the Primrose League present. Its branches will be doing a service to the country at large in coming to the help of charitable movements.

### THE DUTIES OF WOMEN INSPECTORS OF MIDWIVES.

It will be seen from the report of the last meeting of the Central Midwives Board that the Board require the services of a registered medical woman to act as inspector of midwives from time to time. As there appear to be misapprehensions about the duties which persons filling the position of inspectors are expected to perform, we may state that the inspection is limited to the midwife, her bag and appliances, her house and surroundings. It has nothing to do with the treatment of the patients in their own homes.

### THE EXAMINATIONS OF THE CENTRAL MIDWIVES BOARD.

The scheme of examinations issued by the Central Midwives Board has been approved by the Privy Council. The first examination—partly oral and practical, and partly written—will be held in June 1905. Thereafter an examination will be held three times a year, or oftener if necessary, in London and the provinces. The first provincial centres are Bristol, Manchester, and Newcastle-on-Tyne. As to examiners, not fewer than two are to be present, and they are to be men or women who are qualified medical practitioners. But the examiners may, when they see fit, with the consent of the Board, employ for certain parts of the examinations, properly qualified women who are not medical practitioners. The latter are to be remunerated out of the sums paid to the examiners for the examination. The list of examiners to be prepared by the Central Midwives Board is to be subject to annual revision, and applications for the posts are to be invited by means of advertisement. The papers set for examination are to consist of not fewer than six questions, and the time allowed for answering is to be three hours. Notes are to be taken by the examiner or a colleague as to the subjects upon which the candidate is questioned in the oral examination. Fifteen minutes is to be the average period for the oral examination of each candidate, and examiners are requested not to ask candidates where and by whom they have been trained, so as to avoid any suspicion of bias.

### RURAL MIDWIVES ASSOCIATION.

At a meeting of the Rural Midwives Association, held last week at 50 Albemarle Street, Mrs. Heywood Johnstone presided, and gave a short interesting address, in which she said that during the past 18 months 36 midwives of the working-class had been trained and sent out by the Association, but she admitted that there were considerable difficulties in carrying out the Act, especially in sparsely-populated districts. In the discussion which followed Sir Michael Foster described the examination as severe, and expressed his opinion that for the next few years the Association must be content to feel its way slowly; Miss Broadwood complained that the Central Midwives Board was fixing too high a standard; and Mrs. Hobhouse mentioned that in Wiltshire the Association had worked most usefully in connection with the County Nursing Association. It was subsequently resolved that a committee, appointed by the County Council, will be the best authority to carry out the Act; that inspection would be best carried out by a county medical officer of health, or by a fully-trained nurse and midwife under medical supervision, not connected with local nursing associations; and that the best form of rural midwife is a woman with an intimate and practical knowledge of the life of the working-classes.

### MIDWIFERY TRAINING AT THE EAST END MOTHERS' HOME.

The alterations in the training of midwives and monthly nurses at the East End Mothers' Home during the last two years, have been most successful. None but three years' certificated nurses are received for the short course of training, those with no previous training must take the longer course. Out of 52 midwives trained during 1903 and 1904, only two failed to obtain the L.O.S. certificate. The midwife pupils are first taught the management of the mother and baby in the lying-in wards, they then deliver the cases in the labour wards, finishing their training in the district. Gaining excellent experience in every way, they are enabled to get more than the regulation number of cases necessary for the examination. The monthly nurses are trained entirely in the lying-in wards, being allowed to see as many cases as possible. Last year 830 cases were attended, 377 in the home, and 453 in the district. Owing to the increase of the work new and additional quarters had to be provided for the nurses, and each pupil now has a well-furnished cubicle to herself. Lectures are given twice a week by the visiting medical officer and the lady superintendent. The home is recognised as a training school by the Central Midwives Board.

### A BAZAAR ORGANISED BY SHREWSBURY NURSES.

The nurses of the Shrewsbury Nursing Institution, who with the Misses Morgan organised a sale of work in the interests of the Baschurch Convalescent Home for Women and Children, have shown that they can not only discharge their duties efficiently, but also find time to render important assistance to a good cause. The bazaar last week, after payment of all expenses, will bring in about £200, involved many months of hard work, and Lady Bradford, who performed the opening ceremony, did no more than justice to their energy and devotion. Several of the nurses were among the stall-holders, and the promoters of the undertaking must derive satisfaction from the knowledge that some of the patients at the Baschurch Home, where hip disease and tubercular affections are treated entirely out of doors, may be able as the result of a substantial addition to the funds, to remain longer than would otherwise have been possible.

### ENTERTAINMENT AT PADDINGTON CHILDREN'S HOSPITAL.

The annual entertainment at the Children's Hospital, Paddington Green, usually held at the beginning of January, was this year unavoidably postponed, owing to illness among the staff. It took place on Tuesday, when all the wards were prettily decorated with plants and flowers, the former lent by a local tradesman. Fairy lamps and coloured shades added to the effect, and helped to accentuate the contrast presented by the winter gloom outside and the cheerful gaiety within. At three o'clock an excellent programme was gone through in the outpatient department. At five a gigantic tree in the surgical ward was lighted by electricity, and little patients, brought in from other wards, as well as those already occupying the cots, received presents from the visitors. The cost of the entertainment generally was covered by contributions specially collected by the matron, Miss Pinchard, who was assisted in carrying out the arrangements by the sisters and nurses, as well as by the resident medical staff.

### CHILDREN ENTERTAINED BY DISTRICT NURSES.

A very admirable labour of love is undertaken every year by the district staff in connection with the Middlesbrough Nurses' Home, under the auspices of the principal, Miss Purvis. It was started 14 years ago when the home was inaugurated, in the shape of an entertainment to poor children in the town. At first the number of children was so small that the event took place in the house of one of the nurse's patients, but it has gradually assumed larger dimensions, and the gathering which has just taken place taxed the resources of the Town Hall crypt, no fewer than 600 children being present. Some came on crutches, others were wheeled in bath-chairs, and several were carried. Among the waitresses, in addition to the nurses, was the Marchioness of Zetland, who has always taken a great interest in the movement. Lady Zetland, after assisting to dispense tea, helped to distribute the presents from the Christmas tree. One interesting feature was the elaborate dresses of the numerous dolls, which were the gifts of the wives and daughters of poor men; and another the collection of beautiful paper flowers, which were all sent by a cripple girl.

### SOCIAL GATHERING IN SOMERSETSHIRE.

A new centre for the small social gatherings for nurses in Somersetshire has just been organised. The first meeting was held at St. Edmund's Lodge, Glastonbury, on Saturday afternoon, when about 20 nurses and others listened to an interesting and inspiring address from Miss Bristowe, late sister of St. Bartholomew's Hospital, on "The Ethics of Nursing." It may be hoped that good work will be done in this mode of bringing isolated workers together to discuss nursing topics.

### The Nursing Outlook.

"From magnanimity, all fear above;
From nobler recompense, above applause,
Which owes to man's short outlook all its charm."

### WOMEN AND WORK FOR HOSPITALS.

THE discussion which has been proceeding in our columns for some weeks past on the suitability of women for seats on hospital committees reveals the existence of a general desire on the part of many ladies to take some part in hospital work. For the reasons which we stated last week no woman, because she is desirous to be upon a hospital committee, who has never had any special training and who possesses no technical knowledge or experience of business, would be able, if elected, to render any useful service. There are, however, many ways in which women who have leisure and talent can do useful work for hospitals. Many matrons and the chaplains of large hospitals welcome offers of service from such women as visitors in the wards, under definite regulations. As a visitor a lady of discretion may perform many useful offices for the humbler patients and render material service to the executive officers. The circumstances of many patients when ill, in a hospital might be materially alleviated by a visitor who would act as a friend and counsellor in such cases. Illness in such circumstances causes much anxiety to the breadwinner, or to the mother of a family, which may retard recovery until the trouble is lifted by the visitor from the shoulders of the patient. Then, too, many hospital cases, which need convalescent aid or a residence in good air for a brief space after leaving the hospital, could be helped, when otherwise the change, so requisite to speedy recovery, would be impossible.

Again most of the large hospitals have Samaritan funds or guilds worked by women, where good service may be rendered in many ways. The Samaritan funds are devoted to the supply of appliances, grants of money, help for tiding over the period of convalescence, the cost of removal to the country or a convalescent home, and sometimes of a residence at the latter. In all these cases a sensible woman can find opportunity for useful work. It is essential that accurate information should be forthcoming of the circumstances of the patient and the conditions of their families. So the visitor or member of a guild who has become acquainted with a patient in the hospital can visit the home, and in a friendly way gather accurate particulars which must help the hospital authorities and the patient, by enabling the former to provide, on the facts, for the immediate necessities of each case.

The guilds exist to supply clothes for destitute patients on their convalescence or discharge from the hospital. As most of them have been working for a good many years they have gradually undertaken other work in connection with the hospitals, which from time to time has commended itself to the authorities and the executive committees of the guilds. To belong to such a guild is an education, to many who are wholly ignorant of the circumstances and needs of the poor, and as knowledge increases and the desire to more directly help hospital patients grows, a member of a guild may be afforded an opportunity to take her place as a visitor in the hospital, and so to more directly associate herself with individual cases. It will thus be seen that there are several practical ways in which women who have leisure may usefully associate themselves with hospital work, to the advantage of the patients, the assistance of the authorities, and the education of themselves.

Another branch of hospital work has been accomplished through ladies' associations. At the Great Northern Central Hospital, for instance, such an association undertook to raise a large sum for the building fund, and by organising house-to-house collections, sales of work, meetings, and by other means, succeeded in rendering material financial assistance to this institution. When the hospital was completed and opened it was found that the members of the ladies' association were so satisfied with the work they were doing, and so interested in it, that they continued their organisation, and each year they now raise a material sum, which may be regarded as a permanent source of income. The direct benefit in pounds sterling of the ladies' association is considerable, but our experience leads us to feel that its indirect benefit to a hospital may be infinitely greater, for the example set by the working members, who render so large a measure of personal service, has often stimulated their mankind, and so led to the addition of many new names to the list of subscribers, who otherwise might have taken little or no interest in the hospital or its affairs. Wherever we see evidence of a sincere desire to do useful work on the part of women, especially, there we feel called upon to afford every practical assistance in our power to secure that this feeling shall result in practical works for the good of others.

It will be seen that although, as we have shown, there is really no opening at the great hospitals for women on the committees of management, there exists a variety of ways in which women may render useful service to the sick in hospitals, and to their families. In large cities, particularly, women visitors, and those who associate themselves with hospital guilds, do, in fact, render material personal service of the highest value to themselves and to all with whom they may be brought in contact. We hope, therefore, that many recruits may offer themselves for service of this kind, by sending in their names to the matron of the particular hospital on whose behalf they would like to render a little personal service.

### Lectures Upon the Nursing of Mental Diseases.

By ROBERT JONES, M.D.Lond., B.S., F.R.C.S.Eng., M.R.C.P.Lond., Resident Physician and Superintendent of the London County Asylum, Claybury.

### LECTURE XXIV.

IN regard to fractures, they may occur in the insane without showing (1) subjective symptoms, such as pain. The (2) objective symptoms are (*a*) loss of power or inability to move the part or raise the limb, (*b*) deformity, shown by an alteration in shape—*i.e.* by bending or shortening of the limb, (*c*) an irregularity in the course of the broken bone, (*d*) abnormal movement at the seat of fracture, as if there was a joint there, and lastly, the most certain sign, (*e*) *crepitus*—a peculiar grating sensation caused by the rubbing of the two broken ends of the bone against each other. The general treatment of fractures is to bring the portions of the broken bone as nearly as possible into their natural position and to maintain them there, but this is the doctor's work. The nurse has three rules to observe in regard to fractures: (1) Do not handle the patient unnecessarily, so that there should be no risk of a simple fracture becoming a compound one. This can best be attained by moving the patient or the limb as little as possible. (2) Keep the parts at rest. This can be done by applying splints or supports so as to keep the joint above and below the fracture immovable and at rest. The splints should always be outside the clothing and should project beyond the joints above and below the broken bone. (3) Bandages must not be too tight, nor any knots made, nor pressure permitted over the seat of fracture. If there be a compound fracture the first attention must be to the wound; if it is clean, then apply a thick layer of dry lint; but if the wound be dirty, cleanse thoroughly with tepid water that has been boiled, and then apply splints until a special medical examination can be made.

*Dislocation.*—The only duty for the nurse in this case is to keep the patient quiet and to apply a bandage so as to fix the injured part. In bandaging, which is a means of applying even pressure, a few rules are to be observed—viz. (1) Have the outer surface of the roller against the skin. (2) Bandage from below upwards and from within outwards. (3) Let each turn overlap one-half the previous one. (4) The crossings and reverses to be in one line and towards the outside of the limb. (5) The bandage should be securely fixed so as not to slip.

*Unconsciousness.*—The insane are more liable than the sane to suffer from temporary loss of consciousness. The causes of unconsciousness are—(1) Fainting, or syncope, or collapse. (2) Hysteria. (3) Epilepsy. (4) Apoplexy. (5) Shock, or concussion of the brain. (6) Sunstroke. (7) Alcohol, or narcotic poisoning, such as opium. (8) Asphyxia, from choking or drowning.

Fainting or syncopal attacks are always dangerous. The treatment, if the patient is not already lying down, is to lay the patient flat on the floor so as to allow more blood to flow to the brain. The clothing about the neck should be loosened. There should be access for plenty of fresh air. The face may be bathed with water, which should also be given for a drink, and in it a little *sal volatile*, or brandy. If the unconsciousness is due to loss of blood then both legs should be raised to help the return of blood to the heart and brain. Let the head be kept low till consciousness returns.

For hysteria cold water may be applied to the face, but there is rarely danger from these attacks, nor is there complete loss of consciousness. Epilepsy is frequent among the insane in asylums, and is not infrequently a natural termination of some cases of dementia. In the event of an attack care must be taken that the patient does no harm to himself. After the clothing round the neck is loosened place a pillow for the head to rest on. The liability of the patient to bite the tongue can be obviated by introducing an improvised gag. When the fit is over, have the patient carried to a position of safety where he can remain in quiet seclusion, for there is considerable irritability after fits of epilepsy. It is well to give active aperients if the fits continue, and injections of bromide of potassium or bromide and chloral may be prescribed. The convulsions of young children may often be stopped by removing possible causes, such as constipation, indigestible food in the stomach, worms, etc. It is well to try and break the habit of repeating the fits in young persons if possible, by the administration of special medicines, such as the bromides, which have this effect.

Apoplexy is always a serious condition. There is usually loss of power on one side and a heavy stertorous breathing. The patient must be kept quiet. Cold applications to the head and hot-water bottles to the feet are the nurse's duties, and care is necessary to avoid alcoholic stimulants, also a brisk purgative is necessary. Care must also be taken to distinguish the effects of apoplexy from those of alcohol. Concussion is caused by a fall or some injury to the head, and care is again necessary to distinguish the effects of concussion from compression. The patient must be kept quiet, warm, and in a darkened room. For collapse or shock warmth should be applied, a light hot bran-bag over the region of the heart, hot-water bottles to the feet, and easily diffusible stimulants should be given. Sunstroke needs cold applications to the head, with quiet and rest in cool surroundings.

Reference has been made in the lectures to the administration of enemata. These are of several kinds, signified by their use, and are—(*a*) *Astringent* or styptic enemata, such as sulphate of zinc, perchloride of iron and alum; these are used for controlling hæmorrhage. (*b*) *Antipyretic*, such as ice-cold water or solutions of antipyrin, for reducing high fever. (*c*) *Anthelmintic*, such as turpentine, solution of common salt, infusion of quassia-chips, for destroying worms. (*d*) *Antiseptic*—boracic acid, chinosol, quinine, corrosive sublimate, solution of iodine, and Condy's fluid for ulcerative dysentery, or cancerous or other discharges. (*e*) *Sedative*, as starch and water (two ounces), with twenty drops of laudanum for diarrhœa, bromide and chloral for epileptic fits. Suppositories are often used as sedatives. (*f*) *Aperient*, such as soap and water (one to four pints) or this with two ounces of turpentine; olive oil with water; water and sulphate of magnesia (Epsom salts), one to two pints; castor oil (two ounces) and water (one pint); or glycerine (one teaspoonful). (*g*) *Nutrient*, not more than two to four ounces in quantity, of peptonised milk, milk and egg, or beef tea with a tablespoonful of brandy. Artificial feeding in the case of the insane may either be by the mouth, nose (nasal tube), or the rectum. When nourishment by the mouth is forbidden as in gastric ulcers, or rejected as in persistent vomiting, or cannot be administered as in states of collapse, then suppositories of some soluble meat are occasionally used in preference to injections. Enemas should be carefully and slowly administered, the nurse stopping at once if pain is given; and the tube when introduced should be oiled and delicately passed into the rectum, as the patient often resists treatment in this particular. It must be remembered that enemata are either to be retained, when they are termed nutrient, and in this case they must be small; or to stimulate the action of the bowels, in which case the quantity used may amount to several, even six, pints

When the patient uses a commode out of bed a blanket or dressing-gown should always be placed over the shoulders.

The instruments employed for enemata are of various kinds: (*a*) *The glycerine syringe* is made of glass or vulcanite, and it holds any quantity from one teaspoonful (which is the usual dose) to an ounce. (*b*) *The "Higginson syringe,"* which is used for relieving the bowels, and, by attaching a special tube, for vaginal injections. It is simple in use, the bulb being squeezed and released alternately. The fluid flows only in one direction, and as the instrument is usually made of indiarubber the elasticity of the material permits a continuous and even flow. (*c*) *A bulb* or *indiarubber bottle*, capable of containing from four to six ounces, called the "ball syringe," with a short ivory tube screwed on the end of the bottle for administering nutrient enemata, which, as stated, do not exceed four ounces in quantity. (*d*) *The hypodermic syringe*, a small glass tube, with register for measuring drops marked upon it. A fine hollow needle is at one end, and the handle of the piston is at the other. When charged, the contents are injected under the skin.

(*To be continued.*)

### The Nurses of Sussex County Hospital.

### INTERVIEW WITH THE MATRON. BY OUR COMMISSIONER.

If the situation of Sussex County Hospital, right above the town of Brighton and overlooking the sea, is very fine, that of the Nurses' Home which stands many feet higher still, is splendid. Protected by the Downs at the back, the site is an ideal one from a health point of view, and when I had stood in the sunshine on the covered terrace outside, I was not surprised to notice in the wards that pale-faced and weakly-looking nurses were conspicuous by their absence.

### The Nurses' Home.

"How long have you had this delightful Home?" I asked Miss Katharine Scott, the matron of the hospital, as we ascended the heights, choosing the path up the Downs rather than the spacious covered way which connects the two buildings and affords complete protection in inclement weather.

"It was the great feature of the important additions and alterations which have been made during the last ten years as the result of a special appeal by the Committee, and was erected at a cost of between £4,000 and £5,000. The covered way and the bridge connecting the two buildings were a gift."

"Where did the nurses sleep before the Home was built?"

"In the hospital. The need of the Home was urgent; I can speak from personal experience, as I was matron here when the nurses had only very unsuitable rooms to sleep in. At that time, nine years ago although the Committee allowed me to hire two houses near the hospital in order to increase the staff, we could not have as many nurses as we required. The Home has been in the fullest sense of the word a great boon, whether in respect to the health, the comfort, or the discipline of the nursing staff."

### The Value of Separate Bedrooms.

"You do not like cubicles?"

"I regard them as a very serious drawback, and when the plans for the Home were under consideration I made a strong representation in favour of the provision of a separate bedroom for every nurse. It is difficult to exaggerate the value of separate rooms. Nurses want a certain amount of privacy and quiet, which is impossible if they sleep in cubicles. With a room to herself a nurse feels less like a school-girl; while, on the other hand, discipline can be better maintained. For instance, there is no talking at night. Indeed, the regulations are more likely to be observed strictly when each nurse has her own room than when a number of them sleep in cubicles. The entire abolition of cubicles would be a matter for congratulation; and that anyone would propose to perpetuate them in a new home is almost incredible."

"The bedrooms," continued the matron, as we inspected the Home—and I noted that they were admirably furnished, lacking only a fireplace, which is not supposed to be necessary, because they are heated by radiators—"are all alike, and there are about 50. The night nurses have a whole floor

The Nursing Staff of the Sussex County Hospital.

### THE NURSES OF SUSSEX COUNTY HOSPITAL—*Continued.*

to themselves, and are shut off with glass doors. There are four bath-rooms and two box-rooms."

"The sitting-rooms are pretty, and have a charming outlook."

"Yes, they are not large, but they are very comfortable. The nurses go out a great deal and some like to be often in their own rooms. The home sister has a sitting-room to herself and the senior or staff nurses use one of the two general sitting-rooms; juniors and the probationers the other. There is a piano in one of these but not in the other, because it is under the night nurses' quarters and playing would disturb them."

"Are any meals served in the Home?"

"Only breakfast and other meals when a nurse is off duty for a day. This is their great treat, and was not possible before we had the Home."

### Inside the Hospital.

The operating-theatre is on the top floor of the hospital, and when we emerged from the lift the matron said that this was where some of the nurses formerly slept.

"You have now a very light, commodious, and up-to-date operating-theatre?"

"We are very proud of it, and the sister-in-charge, who has a nurse under her, takes pains to keep it in good order. As you will notice, the whole of the building is thoroughly heated, and in the new portions the electric light has been introduced. Moreover, an electric lamp is supplied for each ward, so that all the patients can be examined by the aid of it. The last addition to the hospital was the isolation block, and our next improvement is to be the conversion of the Grant block into a gynæcological block. There is also a very fine pathological department, which is shortly to be opened. This will be effected at the expense of a generous private individual."

"I observe that all the wards appear to be divided into two, and that the space given to each patient is very considerable."

"The latter is quite a feature of the hospital, and as far as possible the beds are placed so that the patients can see the sea from the window. There are 190 beds, and in addition to the large wards there are three outside. Two have 10 beds, which often contain very serious cases, needing a special nurse. The other is a children's ward, with 20 beds. As you know, there is a Children's Hospital in the town, but our ward is generally full. I should not like to be without children, whose care is an important part of the nurse's experience. A sister and three nurses are on duty in this ward by day, and a nurse during the night.

### Paying Probationers.

"How large is your entire staff?"

"There are 46 probationers and staff nurses, 14 sisters, and an assistant matron. The whole of the probationers pay for their training, and receive no salary during the whole of the three years."

"How much do they pay?"

"Ten pounds the first year, and £5 the second. Until two or three years ago the premium was £35. In these circumstances I found a considerable difficulty in obtaining the right nurses, but since the Committee reduced the premium I have had no trouble. My own opinion is that the whole of the probationers should pay premiums, or none of them; and, without reflecting on any nurses, I think that it is easier to maintain a refined tone among paying probationers."

"Do the Committee provide uniform?"

"No; but outdoor uniform is not compulsory. Many find it convenient to wear, but I do not advocate it."

"What is the age of admission?"

"From 22 to 34. Probationers come for three months' trial, which counts in the training, and liberty is reserved to the probationer to leave at the end of the year, or for me to send her away. Of course, a certificate is only given at end of three years, but the power of cancelling the agreement at the termination of one, though it is seldom done, is a mutual advantage. At the expiration of 12 months, I know whether a girl will make a nurse or not, and she is able to judge whether the work suits her. Then, I am allowed to keep on two or three staff nurses at a salary equivalent to £25 a year. One of these staff nurses is responsible for the *x*-ray department. They take holiday duty for sisters and frequently stay to learn massage. One of the sisters trains them and they go direct from here to the examination of the Incorporated Society of Trained Masseuses. Many of our nurses have passed in that way.

### Theoretical and Practical Training.

"I suppose that there are the usual lectures?"

"During the first year the matron lectures, and during the second and third, either one of the honorary staff, or the house surgeon or physician, lectures on medical and surgical subjects, an examination taking place at the end of each course of lectures. As to the hours of duty all probationers and staff nurses breakfast at 6.45 and are on duty at 7 A.M. Half an hour each is allowed for lunch, dinner, and tea. Supper is at 8.30 and prayers are at 9. All meals are served in the dining-hall. The probationers are off duty for two hours every day and often more. Twice a week the staff nurses go off at 6 P.M. The hours are long, but I do not see how they can be shortened."

"What about holidays?"

"They are entitled to three weeks and two days at the end of each year. But they have also a day off once a month and can always get away the night before if they wish to. They have night duty for three months at a time, and instead of having one night off occasionally, they have five days off when the three months are up."

"How long are the night nurses on duty?"

"From 8.30 A.M. to 8.40 P.M. They are free at 9.30; but they must have seven hours in bed."

### Difficulties of Diet.

"Do you have any difficulties in respect to diet?"

"Every matron, I think, finds the night meals a worry. There is no trouble about breakfast or dinner. But I give them as much variety as possible in food and they always have tea or coffee. There are 14 or 15 night nurses and a sister-in-charge. Two nurses are allotted to the two medical wards and two to the largest surgical wards, and to the other wards as occasion requires."

"What salaries do the Sisters receive?"

"They start with £37 10s. and go up to £42 10s., increasing £2 10s. a year. Nearly all our sisters were trained here. They are on duty at 8. They breakfast together, and have dinner and supper at their own table, but at the same time as the nurses. Tea is served them in their own room. They are off duty four evenings at 6, and on two they have the entire afternoon; they also have a free evening twice a week, on which evenings the staff nurses give in the reports. This relieves the sisters and imposes a salutary responsibility on the staff nurses. They are off duty alternate Sundays from 10.30 to 1, and they have a holiday of a month and two days. The nurses always attend one or two of the three services in the chapel on Sunday at 7.45, 9.30, or 5.30." The nurses need not be members of the Church of England. There is no sort of religious test. We have two or three Roman Catholics whom we allow to attend their own churches, and some Nonconformists, who do not object to attend service in the chapel."

THE PRIVATE STAFF.

"Are you responsible for the private nursing staff?"

"Entirely. The two houses in which they reside in Eastern Road are in charge of a home sister, and she does the housekeeping. There are 30 private nurses. They were not all trained here, but I hope to have more of our own nurses in time. The salary paid is £40, and they have in addition a bonus, which is always as much as £15. At the end of four years the salary rises to £50, and with bonus is £65. They provide their own uniform, which is obligatory."

"Are their services greatly in demand?"

"Yes. But I have as many as I can thoroughly know. I never take outside nurses without special recommendation, and I never advertise. They frequently go long distances. I have one at Swansea just now; but most of them work in Brighton and in the country round."

PENSIONS.

"Is there any provision made in respect to pensions for the hospital staff?"

"No, not at present; but the committee are very good to all the nurses when they are ill. The Sisters and private nurses get full pay for three months, and half-pay for three more. Every private nurse who falls ill is brought into the hospital. A good many of both the hospital sisters and private nurses belong to the Royal National Pension Fund, and I think all should, but it is difficult to induce some to join."

"I think you mentioned that you had been here nine years?"

"Nine years in February. I was trained here; and, except a short interval, during which I had charge of a small hospital I have been at the Sussex County Hospital the whole of the 16 years in which I have been engaged in nursing."

### Central Midwives Board.

A MEETING of the Central Midwives Board was held at the Board Room on Thursday last week. There were present: Dr. Champneys (chairman), Dr. Cullingworth, Dr. Ward Cousins, Miss Paget, Miss Wilson, and Mr. Parker Young.

Amongst the correspondence before the board was a letter from the Clerk of the Privy Council, approving the Board's scheme of examinations. It was agreed that the first examination should be held during the last week of June, the schedules to be sent in by June 1st.

POOR-LAW TRAINING SCHOOLS.

A number of memorials from the Boards of Guardians of Bradford, Leeds, Blackburn, etc., "praying the Board to reconsider their decision not to recognise Poor-law training schools as such," were presented by the secretary. The Board approved of the replies already given by Mr. Duncan to the memorialists, pointing out that no such decision had ever been come to by the board, several Poor-law institutions having been already recognised and each case considered on its merits. The secretary drew attention to the fact that several institutions represented in the memorials had never applied for approval.

Miss Wilson proposed that the whole Poor-law question be considered at a special meeting, and the following resolution was seconded by Dr. Cullingworth and carried:—

"That a special meeting be held to consider the principles which should govern the approval of Poor-law institutions as training schools."

It was pointed out that Poor-law infirmaries, coming, as they did, under a State department, were in a different position to any other institutions with which the Board had to deal.

INSPECTORS OF MIDWIVES.

Letters were read from various supervising authorities and medical officers of health with reference to midwives practising in their areas. Consideration of these was postponed, pending further information. In reply to a letter from the secretary of the London School of Medicine for Women, asking the Board to recommend local supervising authorities to appoint only qualified medical practitioners as inspectors of midwives, the secretary was instructed to say that the Board had no power to make such a recommendation.

After other letters had been dealt with, the following resolution, moved from the chair, seconded by Dr. Cullingworth, was agreed to *nem. con.*:—

"That all matters relating to applications for recognition, appointment as examiner, or other matters of which the discussion of persons forms a necessary part, be considered by a Standing Committee of the whole Board, and the conclusions reported to the Board."

Miss Wilson then moved: That the following advertisement be issued by the board:—

"Midwives Act, 1902 The Central Midwives Board requires the services of a registered medical woman who has acted as house surgeon or physician in a maternity hospital or home, possessing an extern department, to undertake, from time to time, the duties of inspector at a fee of £1 1s., with travelling expenses, for each inspection. Particulars and form to be obtained from the secretary."

This was seconded by Dr. Cullingworth and carried, after considerable discussion, with one dissentient—Mr. Parker Young—who was of opinion that the appointment should be open to medical men. Miss Paget considered that in many respects a trained nurse and midwife would be the best choice for such inspection, much of which referred to practical nursing details. Both she and Miss Wilson were of opinion that a medical woman, with such qualifications as those indicated, would have greater experience in the actual practical training of midwives than medical men.

The rest of the business was adjourned to February 9th when it will be taken after a special meeting, called to consider the question of Poor-law Training Schools.

### Nurses in Court.

IN the case of Cowan *v.* Morell, heard before Mr. Justice Jelf in the King's Bench Division, and concluded on Wednesday, both the plaintiff and defendant were described as nurses.

Mr. Wildey Wright, in opening the case for the plaintiff, said that the defendant, while denying slanders, admitted that she wrote the letters described as libels, but pleaded that they were true in substance and in fact. She further pleaded privilege and justification. The plaintiff was the daughter of a major in the Army; and, having gone through a hospital training, had opened a nursing home at Putney, where she received patients, and whence she sent out nurses. Three sisters, Ada Thirza, Alice, and Fanny (known as Daisy) Morell, also supplied nurses, charging a commission for doing so. They did not receive patients, but two of them nursed patients in their own homes. In December 1902 a dispute arose concerning a commission claimed by the defendant in respect of a case at Haslemere, to which a Nurse Hawker and subsequently Nurse Bland went. The plaintiff refused to pay, and the defendant threatened to injure her in her business. About two months later a Mrs. Groome made inquiries of Miss Morell concerning Miss Cowan's home, and the letters complained of were sent in reply. Mr. Wright contended that libels and slanders of this nature, widely disseminated in the neighbourhood where the plaintiff resided, had a very bad effect on trade. The purpose of the action was to stop them, to prove that his client was not the immoral woman she was represented to be, and to reinstate her character.

Mrs. Groome, who said that she had a registered home, under inspection by the County Council, at Gateley Road, Brixton, stated that in making inquiries about nursing homes she used an assumed name and address, because nursing homes were so bigoted one against another, that if this were not done inquiries would be ignored. It was the same with monthly nurses. In this case, she was not even sure if the Morells were really nurses. Not every one who put on the uniform was a nurse.

Miss Mary Cowan said that she was a qualified and certificated nurse, having diplomas and testimonials from several hospitals, principally St. Mary's, Paddington, St. George's, and Queen Charlotte's Lying-in Hospital. She had followed the occupation of a nurse for upwards of 10 years. She denied the accusations brought against her. She did not know the defendants until October or November 1902, when Alice Morell applied to her for work, and was given a case, which she left without permission. The accusations had caused her a great deal of injury in her business.

Mrs. Maude, a patient of Miss Cowan, said she engaged her, after assuring herself that she was a certificated midwife, notwithstanding allegations made by Miss Morell, whom, as a district nurse, she at first consulted. Miss Cowan nursed her very well, and she had promised to speak on her behalf if occasion arose.

Mr. Atherley Jones, on behalf of the defendant, addressed the jury for over an hour, taking the view that there was a deliberate attempt to damage the character of his client by a cruel fabrication. He contended that she had acted within her province as a district nurse, and added that in neither case complained of was the plaintiff's business injured, since both Mrs. Groome's "foster daughter" and Mrs. Maude became her patients, notwithstanding the alleged libels and slander. He held that a detective policy had been pursued in order to get up a case. He dismissed the assertion as to the bigotry of one nursing home towards another as moonshine. As a matter of fact, there was no inspection of nursing homes.

Miss Morell, who described herself as a salaried district nurse, who added to her earnings by taking patients who paid small fees, admitted that she had latterly charged commissions to nurses for whom she procured patients, but denied any altercation with the plaintiff as to commission on the Haslemere case. She had been a nurse for about 14 years, and had been at Paddington and Richmond Infirmaries, Marylebone Children's Home, and the South Western Nursing Institution.

Nurses Hawker and Bland denied that money was owing from them to Miss Cowen, and stated that they were unable to get from her what was due to them in fees.

The jury returned a verdict for the defendant, and judgment was given accordingly.

### Everybody's Opinion.

### NIGHT DUTY IN SMALL WORKHOUSE INFIRMARIES.

"POOR-LAW NURSE" writes: I should like to say a word or two about Nurse Eva's letter. I have had many years' experience, and twice in small Workhouse Infirmaries, with over 30 patients, and two nurses on day duty, yet in each case it was thought most unnecessary to have a night nurse, although many of the patients were quite helpless, and needed night care. But unless actually dying, a nurse was not expected to sit up, and then she had to manage without extra help, although she might have to sit up several nights in succession. Happily, I had a reasonable matron to work under, and was able to take a rest during the day. Previously a nurse had never had the courage to ask to go off duty, but not being strong, I could not work night and day without breaking down. I can hardly understand a nurse being faint-hearted, as if she really loves her work, she will never give up while she has strength to do her duty. Unfortunately, I cannot work hard for more than two or three hours without feeling tired, but I have learnt that it is wiser to keep the fact to myself, and during the afternoon I can usually take it quieter, and so am ready for the rush again at night. Then there are at least twelve hours off duty, and I am fresh for another day's duties. I should like to advise Nurse Eva to take a rest and try again in a larger union infirmary, where, in addition to more comfort, there are regular hours and alternate day and night duty, which is far less trying than being constantly disturbed at night.

### Appointments.

BOLTON INFIRMARY.—Miss Isabella Milne has been appointed sister. She was trained at the Royal Hospital for Sick Children, Edinburgh, and Leeds General Infirmary.

GENERAL HOSPITAL, NOTTINGHAM.—Miss Mary H. Kemp has been appointed sister. She was trained at the Children's Hospital, Glasgow, and the Royal Infirmary, Dundee.

GLASGOW LOCK HOSPITAL.—Miss Eliza Weale has been appointed charge nurse. She was trained at Bradford Union Hospital, where she was afterwards charge nurse. She has since been nurse at the Manchester and Salford Lock Hospital, and charge nurse of the Lock Wards at the Royal Portsmouth Hospital. She has also been on the private staff of the latter institution. She holds the L.O.S. certificate.

GRAVESEND HOSPITAL.—Miss Kathleen Pomeroy has been appointed night sister. She was trained at St. Marylebone Infirmary.

ISOLATION HOSPITAL, GRENOSIDE, NEAR SHEFFIELD.—Miss Jeannie Booth has been appointed matron. She was trained at the Royal Infirmary, Preston, and has since been night sister at the City Hospital, East Liverpool, in charge of a temporary hospital for enteric cases at Liscard, temporary sister at the Plaistow Fever Hospital, London, and sister at the Private Surgical Hospital, Cardiff.

KEIGHLEY UNION INFIRMARY.—Miss Charlotte Seymour Yapp has been appointed charge nurse. She was trained at Aston Union Infirmary.

LONDON OPEN-AIR SANATORIUM, WOKINGHAM. — Miss Elizabeth Murton Philpott has been appointed staff nurse. She was trained at Bromley Cottage Hospital and King's College Hospital, London. Before her training at King's College Hospital she was assistant nurse at St. Bartholomew's Convalescent Home, Swanley.

MILL LANE FEVER HOSPITAL, LISCARD, CHESHIRE.—Miss Elsie Hill has been appointed sister. She was trained at the City of Dublin Hospital, where she was afterwards staff nurse, and on several occasions discharged the duties of sister.

MINSTER INFIRMARY.—Miss Annie Clough has been appointed assistant nurse. She was trained at St. Peter's Home, Kilburn, by the Meath Workhouse Nursing Association.

OLDHAM INFIRMARY.—Miss Amy Erwood has been appointed theatre and home sister. She was trained at King's College Hospital, London, after which she worked on the staff of the London Nurses' Co-operation. She has since been sister at Lincoln County Hospital, and sister of male wards at Oldham Infirmary.

POPLAR AND STEPNEY SICK ASYLUM (BLACKWALL BRANCH ASYLUM).—Miss Jeannette Grellier has been appointed temporary staff nurse. She was trained at the Cancer Hospital, Brompton, and has since been nurse at the Horton Asylum, Epsom.

QUEEN ALEXANDRA'S ROYAL NAVAL NURSING SERVICE. Miss Florence Mary Leale has been provisionally appointed nursing sister. She was trained at Huntingdon County Hospital, and Bristol Royal Infirmary. She has since been staff nurse at Ealing Cottage Hospital, and nurse-in-charge of the Infirmary at the Royal Naval School, Eltham.

### Where to Go.

ANDERSON, ANDERSON, AND ANDERSON, LIMITED. Great annual stocktaking sale, February 1st to 28th, at 37 Queen Victoria Street, E.C., and 58 and 59 Charing Cross, S.W.

### Echoes from the Outside World.

### Movements of Royalty.

The King unveiled on Saturday, in Holy Trinity Church, Windsor, the Guards' memorial to the 742 officers, non-commissioned officers, and men of the Household Brigade who lost their lives in the South African war. The memorial consists of a series of commemorative panels which occupy the entire length of the wall of the church. At the east end of the south wall is a tablet bearing an inscription, the names of all whose memory is commemorated being given. His Majesty was accompanied by the Queen, and preceded by the clergy, and followed by the military officers, they passed up the central aisle of the church to their seats. In performing the ceremony the King, turning to face the congregation, said, "To the glory of God, and in honoured memory of the officers, non-commissioned officers, and men of the Household Brigade, who fell in the South African war, I unveil this memorial." At the conclusion of a short service the congregation sang a verse of the National Anthem to the accompaniment of the military bands.—In consequence of the illness of Princess Victoria the King postponed his visit to Lord Rosebery, and the Queen came from Windsor to town instead of going to Sandringham on Monday.

### The Situation in Russia.

St. Petersburg has now resumed its wonted appearance, and General Trepoff, the military governor, is confident that he will be able to maintain order. There are still, however, large numbers of men on strike elsewhere, and at Warsaw there was a serious collision between the strikers and troops on Saturday. The city has since been converted into a military camp.—The British Ambassador at St. Petersburg having made representations to the Russian Government respecting placards posted up in Moscow and Libau accusing Great Britain of supplying funds for the workmen's revolt, has been assured that the matter shall be inquired into, and steps taken to prevent any repetition of such publications.—The British Consul-General and Vice-Consul at Warsaw have been attacked, and the British Military Attaché in St. Petersburg is making an investigation on the spot.

### The War in the Far East.

According to reports from Russian and Japanese sources fighting is still going on between the westerly wings of the forces fronting one another in Manchuria. The Russians are said to have advanced 65,000 men, and to have more artillery in action than in any previous conflict in history. The weather is bitterly cold, and the operations are intermittently hidden by heavy storms of snow. The Russians claim certain successes, but Marshal Oyama's despatches are to the effect that they have everywhere been repulsed. He reports the capture of 500 prisoners.—One of the Tsar's private secretaries, in reply to a letter by an English correspondent suggesting that the war might be ended by arbitration, writes that the time has not come for talking about peace conditions. Anything like interference would, he adds, arouse the greatest antipathy in Russia.

### The Largest Diamond in the World.

A diamond weighing 3,030 carats was found at the Premier Mine, Johannesburg, on Thursday last week. The stone is of excellent quality, but of irregular form, and it is the biggest diamond that has ever been discovered. Lord Milner has congratulated the finder. The Premier Mine is situated near Pretoria and has been opened up since the conclusion of the war. If present indications are fulfilled the mine will produce an enormous income for the Transvaal revenue, the particular feature of it being that under the operation of the new Diamond Law, six-tenths of the stones are held by the Transvaal administration. The largest diamonds found previously were Excelsior, discovered in South Africa; the Great Mogul, Regent, Orloff, and Koh-i-nor, discovered in India. Of these, the Excelsior, the latest found, weighing 971 carats, was valued in its natural state at a million sterling.

### The New R.A. and Associates.

Mr. David Murray has been elected an Academician, in succession to the late Mr. Val Prinsep; and Mr. David Farquharson and Mr. Reginal Blomfield Associates. Mr. Murray, who is a native of Glasgow, and was born in 1849, is a landscape painter of distinction and power. His first contribution to Burlington House was in 1875. At that date he resided in Scotland, and for some time he painted scenery of the Highlands. In 1882 he moved to London, and turned his attention to English scenery. Two of his pictures, "My love is gone a-sailing," and "In the country of Constable," were purchased by the administrators of the Chantrey Fund. Mr. Farquharson is also a native of Scotland, and was born in Perthshire about 1830. He [illegible] Associate of the Scottish Academy, but has lived in London since 1884. His picture, "Full Moon and Spring Tide," a recent piece, was hung in the Academy last year in the place assigned by custom to a work by the President. Mr. Blomfield was born in Kent in 1856, and is a grandson of Bishop Blomfield. He has built a great number of country houses, and is also well known as a writer on architecture.

### Lady Wimborne's Bookshop.

On the 16th of this month "The Church of England Bookstore" will be opened in Dover Street, Piccadilly. The undertaking is that of Lady Wimborne, who is both the originator and the capitalist. Her avowed primary object is the sale of Protestant, theological and devotional works, and she states her conviction that in offering such literature for purchase, her establishment will supply a long-felt want. Lady Wimborne adds that she has tried to make her shop attractive and thoroughly up to date.

### Pauper Children for Canada.

A conference of members of Boards of Guardians in London and the provinces with other persons interested in Poor-law questions was held at the Mansion House on Monday to discuss the scheme of Mrs. Close for sending children now chargeable to the rates to farm homes in New Brunswick and Nova Scotia. A resolution requesting the Local Government Board to consider the scheme was declared by the Lord Mayor to be carried, but there was a very strong opposition, and several speakers, including Dr. Barnardo, condemned it as absolutely unworkable. Mrs. Close herself stated that throughout a recent tour in Canada she met with universal enthusiasm for her proposal.

### Fire at a Telephone Exchange.

On Friday evening, shortly after six, the Exchange of the National Telephone Company in London Wall was the scene of a destructive fire. Upwards of 300 girls are employed at the Exchange, and though many had left the premises, a considerable number were still on duty. The fire originated in the principal room on the second floor, where a similar outbreak occurred over a year ago, and the cause is stated to have been the fusing of one of the wires by a gas-stove. The room was soon alight, and two operators who were engaged there rushed into the switch-room adjoining, which was filled with operators, to warn them to leave the premises. Without waiting for hats or jackets they quickly got out of the building, and crossed to Finsbury House, where they unofficially called the muster-roll, all being safely accounted for.

### Notes and Queries.

**REGULATIONS.**

**The Editor is always willing to answer in this column, without any fee, all reasonable questions, as soon as possible.**

**But the following rules must be carefully observed:—**

1. **Every communication must be accompanied by the name and address of the writer.**
2. **The question must always bear upon nursing, directly or indirectly.**

**If an answer is required by letter a fee of half-a-crown must be enclosed with the note containing the inquiry.**

*Excessive Perspiration.*

(134) Will you please tell me what is the best treatment for hands that perspire too freely? For massage this is an enormous drawback, the hands feeling cold and clammy, or else hot and wet. Besides for ordinary occupations, it is an uncomfortable condition at all times.—*L. L.*

For local treatment of the trouble try boracic powder. The root of the difficulty probably lies in deficient general health, for the remedy of which you should consult a medical man.

*Training.*

(135) I am just nineteen and very much want to become a hospital nurse. Can you tell me if any hospital requires a probationer of my age, although I am afraid I am too young?—*T. M.*

At your age you could only be accepted as probationer in a children's hospital. See "The Nursing Profession: How and Where to Train."

I am 36 years of age and wish to train as a probationer in a hospital near but out of London. Do you think my age would be any difficulty? I could not pay any premium but would give a few months without a salary.—*Energy.*

You will find it difficult, at your age, to train in a good hospital, unless you are a paying probationer. But see "The Nursing Profession: How and Where to Train," which will give you every information.

*California.*

(136) Will you kindly inform me if there is any demand for nurses in California either for private or district work, and what is about the cost of passage out? I have a certificate from this infirmary, and have already a three years' certificate for fever work.—*Emigrant.*

Have you the L.O.S. certificate? You would find it difficult to get district or private work in California without it. The Colonial Nursing Association might be able to help you. Write to the Hon. Sec., Imperial Institute, S.W. Also write to the secretary of the United British Women's Emigration Association, Imperial Institute, S.W., and to the Hon. Sec. of the Girls' Friendly Society Emigration Department, 39 Victoria Street, S.W.

*Boston and New York.*

(137) Can you give me the names of any nursing associations in Boston and New York, where male nurses are employed?—*Reader.*

Male nurses are employed at the Roosevelt Hospital, 59th Street and 9th Avenue, New York. You might write to the directress of nurses.

*Zostera Mattresses.*

(138) Can you please tell me whether mattresses of zostera or sea-wrack (commonly called alva) are obtainable? and, if so, where?—*Matron.*

They are obtainable, but to special order only, from Whiteley's, Westbourne Grove, W.

*Sponging and Infection.*

(139) 1. Do most authorities teach that patients should be dried after wet sponging? The recent competitive answers appear to imply this, but I was taught to leave the patient to get dry. 2. Are diseases infectious during the incubation period?—*Hey What.*

1. The point is a disputed one. But it is usual to gently and quickly dry the patient after sponging. 2. Yes.

**Handbooks for Nurses.**

| | Post Free. |
|---|---|
| "The Nursing Profession: How and Where to Train" ... | 2s. 4d. |
| "The Nurses' Pronouncing Dictionary" ... ... ... | 2s. 0d. |
| "Nursing: its Theory and Practice" (Lewis) ... ... | 3s. 6d. |
| "Surgical Bandaging and Dressings" ... ... ... | 2s. 0d. |
| "A Complete Handbook of Midwifery" (Watson) ... | 6s. 4d. |

Of all booksellers or of The Scientific Press, Limited 28 & 29 Southampton Street, Strand, London, W.C.

### For Reading to the Sick.

THE DIVINE PROMISE.

O JESUS, Thou hast promised
To all who follow Thee,
That where Thou art in glory
There shall Thy servant be;
And, Jesus, I have promised
To serve Thee to the end;
O give me grace to follow,
My Master and my Friend.

O let me see Thy footmarks,
And in them plant mine own;
My hope to follow duly
Is in Thy strength alone;
O guide me, call me, draw me,
Uphold me to the end;
And then in Heav'n receive me,
My Saviour and My Friend.

*J. E. Bode.*

Our Lord asks His second question: "So long a time have I been with you, and have you not known Me?" "So long a time have I been with you," and never done you any harm, but much good; "Have you not known Me? Hath there stood One in the midst of you whom you know not?" Friends use this formula to one another at times; "You don't yet know me," they say. Our Lord has deigned to call us His friends. Have we then been these many years with Him, and do we not yet know Him? He has His own ways, which puzzle us at times; all seems strange and unaccountable; but can we not trust Him always to be about His Father's business, always to be working what is best in His sight and best for our eternal welfare? Shall not our part be to follow Him loyally day by day, to cling to Him through all the darkness, knowing how true He has ever been, and that at the last, if faithful till death, He will lead us home?

O my Master, my truest Friend, have I ever doubted Thee or seemed to doubt Thee? Too late have I known Thee, too late have I loved Thee. I renew my sincere confidence in Thee, and on it I will rest, offering it to Thee as my truest worship. I thank Thee with all my heart for speaking to me so well and openly; the whole way to heaven is lit up by Thy saving word; never again let me strike Thee by my sin or disobedience, but let me heal Thy wounds and stem the flow of Thy Precious Blood by my daily service of Thee. And if, dear Lord, at times Thou seemest to withdraw Thyself from me, or to ask of me more than usual, or to lead me by darksome ways and hidden paths most strange to me: even then I will cling to Thee in trustful love, that having been so long a while with Thee, I may know always that it is Thy hand that guides me by the best of roads helping me till death shall come and my eyes shall be opened, till I shall see Thee, my truest Friend, face to face, on the eternal shore.—*Anon.*

Our restless spirits yearn for Thee,
Where'er our changeful lot is cast:
Glad when Thy gracious smile we see,
Blest when our faith can hold Thee fast.

O Jesus, ever with us stay;
Make all our moments calm and bright;
Chase the dark night of sin away
Shed o'er the world Thy holy Light.

*From the Latin.*

## Figures and Tables

**Figure f1:**